# Control of Foot-and-Mouth Disease during 2010–2011 Epidemic, South Korea

**DOI:** 10.3201/eid1904.121320

**Published:** 2013-04

**Authors:** Jong-Hyeon Park, Kwang-Nyeong Lee, Young-Joon Ko, Su-Mi Kim, Hyang-Sim Lee, Yeun-Kyung Shin, Hyun-Joo Sohn, Jee-Yong Park, Jung-Yong Yeh, Yoon-Hee Lee, Min-Jeong Kim, Yi-Seok Joo, Hachung Yoon, Soon-Seek Yoon, In-Soo Cho, Byounghan Kim

**Affiliations:** Animal, Plant and Fisheries Quarantine and Inspection Agency, Anyang, South Korea (J.-H. Park, K.-N. Lee, Y.-J. Ko, S.-M. Kim, H.-S. Lee, Y.-K. Shin, H.-J. Sohn, J.-Y. Park, J.-Y. Yeh, Y.-H. Lee, M.-J. Kim, Y.-S. Joo, H. Yoon, S.-S. Yoon, I.-S. Cho, B. Kim);; Univeristy of Incheon, Incheon, South Korea (J.-Y. Yeh)

**Keywords:** foot-and-mouth disease, foot-and-mouth disease virus, FMD, FMDV, hoof-and-mouth disease, South Korea, diagnosis, herpangina, Picornaviridae, Aphthovirus, viruses, vaccination, epidemic

## Abstract

An outbreak of foot-and-mouth disease caused by serotype O virus occurred in cattle and pigs in South Korea during November 2010–April 2011. The highest rates of case and virus detection were observed 44 days after the first case was detected. Detection rates declined rapidly after culling and completion of a national vaccination program.

Foot-and-mouth disease (FMD) is a highly contagious disease caused by foot-and-mouth disease virus (FMDV; family *Picornaviridae*, genus *Aphthovirus*). FMDV serotypes O, A, and Asia1 are widespread in Southeast Asia (*1*). In South Korea, small-scale outbreaks of FMDV infection caused by serotype O occurred in March 2000, May 2002, and April 2010 (*2*–*5*), and an outbreak caused by serotype A occurred in January 2010 (*6*). In contrast, an outbreak during November 2010–April 2011 was much more widespread (*7*). We reviewed the progression of this outbreak and methods used to control it, including culling and vaccination of pigs and cattle.

## The Study

Clinical signs of FMD in animals appeared on November 23, 2010, in a pig-farming complex in Gyeongbuk Province. Reporting to the central government was delayed for ≈1 week because of misdiagnosis caused by false-negative results from a pen-side antibody kit. FMD-positive test results were confirmed on November 28–29 (Table 1) in samples from saliva, vesicles, and detached hooves from pigs with signs typical of FMDV infection (i.e., salivation, vesiculation, and ulceration) (*7*). Samples from pigs with clinical signs of infection tested positive by antibody-detection assay using solid-phase competition ELISA (PrioCHECK; Prionics, Schlieren, Switzerland) for the O serotype, excluding liquid-phase blocking ELISA; however, antibody tests using nonstructural protein ELISA (VDPro; Jeno Biotech Inc., Chuncheon, South Korea) showed negative results (Table 1).

**Table 1:** Laboratory diagnosis of FMDV infection in specimens from a pig-farming complex, Gyeongbuk Province, South Korea, November–December 2010

Farms in pig complex	Date of sample collection	Antigen detection			No. animals tested	No. antibody-positive animals	
		Specimen type	RT-PCR	Antigen ELISA		SP-O ELISA	NSP ELISA
A	Nov 28	S, V, H	+	O serotype	10	2	0
B	Nov 28	S, V, H	+	O serotype	10	2	0
A	Nov 29	Serum	+	ND	90	3	0
B	Nov 29	Serum	+	ND	40	1	0
C	Dec 1	Serum	–	ND	20	0	0
D	Dec 1	Serum	–	ND	20	10	0
E	Dec 1	Serum	–	ND	41	17	7

*FMDV, foot-and-mouth disease virus; RT-PCR, reverse transcription PCR; SP-O, structural protein of FMDV serotype O; NSP, FMDV nonstructural protein; S, saliva; V, vesicle; H, detached hooves; +, positive; ND, not done; –, negative.

**Figure 1:**
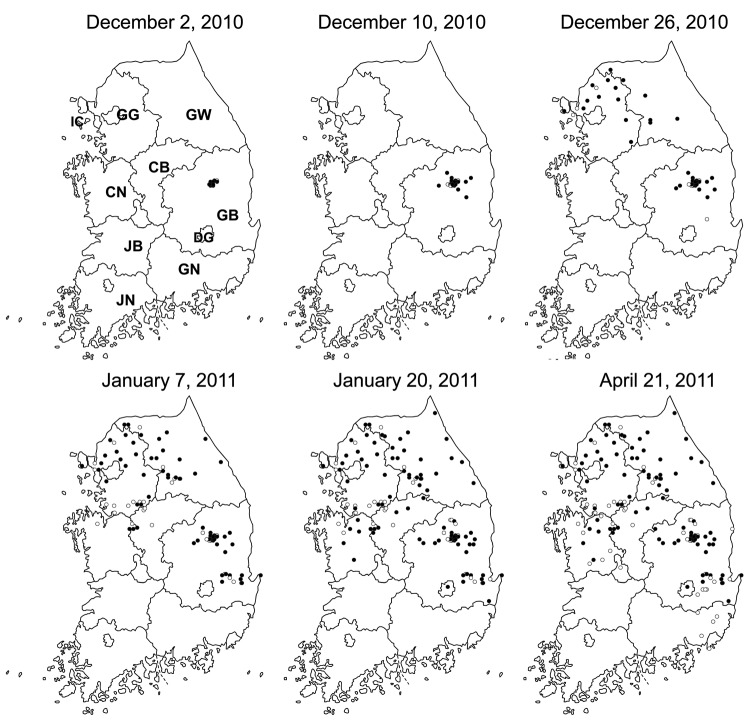
Progress of foot-and-mouth disease transmission throughout South Korea during 2010–2011 outbreak. Circles indicate cases in swine at index farms; black dots, cases in cattle. A timeline of case detection is provided in online Technical Appendix Figure 1 (wwwnc.cdc.gov/EID/article/19/4/1-1320-Techapp1.pdf). IC, Incheon; GG, Gyeonggi; GW, Gangwon; CN, Chungnam; CB, Chungbuk; GB, Gyeongbuk; GN, Gyeongnam; JN, Jeonnam; JB, Jeonbuk; DG, Daegu.

Because many cattle farms were located in the areas surrounding the pig-farming complex, the virus was detected mainly in cattle during the next 25 days (Technical Appendix Figure 1). After the first detection, the disease spread to 75 cities or counties in 11 provinces over 144 days, through April 21, 2011; the only provinces not affected were Jeonbuk, Jeonnam, and Jeju (Figure 1). As soon as an outbreak was reported, animal movement restrictions were imposed, and a 3-km radius protection zone and 10-km radius surveillance zone were set around the outbreak area. 

FMD spread throughout Gyeongbuk Province until December 14; at the same time, it spread rapidly to other regions, including the provinces of Gyeonggi (December 15), Gangwon (December 21), and Incheon (December 23) (Figure 1). For emergency disease control, vaccines were initially administered to cattle in these outbreak areas on December 25. However, FMD continued to spread into additional provinces during January 2011, with outbreaks occurring in Chungnam (January 1), Chungbuk (January 3), Daegu (January 17), and Gyeongnam (January 24). Nationwide vaccination was implemented on January 13, and the last reported case occurred on April 21 in Youngcheon City, Gyeongbuk Province. 

During this outbreak, 153 (73.56%) of 208 farms with suspected cases were confirmed as index points for disease transmission into new areas. Of farms with animals showing clinical signs, 3,234 (83.98%) of 3,851 had positive test results for FMDV in animals. Within the affected areas, after FMDV infection was confirmed in 1 farm, 295 (21.24%) of 1,389 other farms had positive test results. For farms related to the infected farms epidemiologically (e.g., by vehicle movement or human contact), 33 (10.68%) of 309 had positive test results.

An FMD vaccine of high potency was imported for emergency vaccination; the vaccine used FMDV strain O1 Manisa (*8*). A postvaccination analysis using serum samples collected from vaccinated animals and viruses isolated in the field showed the vaccine’s high efficacy in the field. Cattle in the affected regions were vaccinated first, on December 25; later, vaccination was expanded to the whole cattle population, with vaccination completed by January 31, 2011. Pigs were vaccinated 14 days after the cattle (January 8), and the whole pig population was also vaccinated by the end of January. 

According to national policy, culling began in November 2010 for all animals on farms with infected animals. Once vaccination was expanded nationwide in mid-January 2011, a vaccination-to-live policy was implemented; that is, vaccinated animals on farms with infected animals were culled only if the outbreak began within 2 weeks after vaccination but not if the outbreak began >2 weeks after vaccination. Most culled animals were disposed of by burial, which was regarded as a suitable method for a large-scale outbreak, given its advantage of easy handling within a short time. Approximately 3.48 million animals (151,425 cattle, 3,318,299 pigs, 8,071 goats, and 2,728 deer) were buried at 4,583 burial sites (Technical Appendix Figure 2). Some farms that were required to cull livestock because of FMD risk did not undertake the process in a timely manner, which contributed to a spike in new infections on the 38th–64th days after the outbreak began (January 4–31, 2011) (online Technical Appendix Figure 3). These new infections, mainly among pigs, occurred in Chungnam, Chungbuk, Gangwon, Gyeongnam, and Gyeonggi provinces.

After vaccination and culling were implemented, the number of daily FMD cases decreased gradually. Among cattle, the number of FMD cases began to decrease on the 40th day after the initial outbreak (12 days after the first cattle vaccinations). In pigs, the number decreased after the 60th day (18 days after the first pig vaccinations) (online Technical Appendix Figure 1). Many animals also were culled during January 2011 (Technical Appendix Figure 2), and the number of FMD outbreaks decreased to as low as a single index case daily after January 31, 2011.

**Figure 2:**
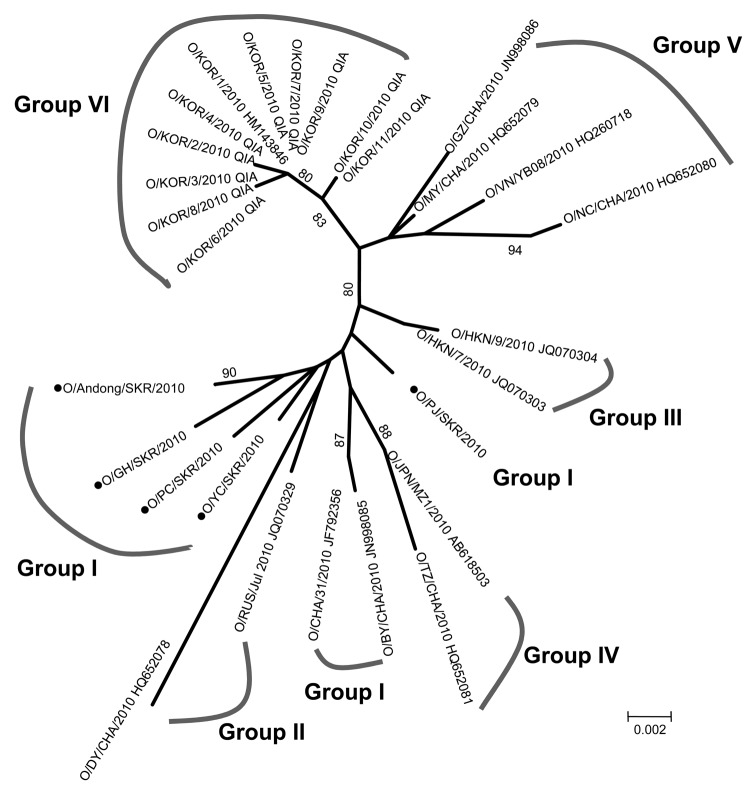
Phylogenetic analysis of viral protein 1 sequences of serotype O foot-and-mouth disease viruses isolated in South Korea (black dots) and other Asian countries, 2010. The tree was constructed by using the neighbor-joining method in MEGA5 (www.megasoftware.net). Percentages in which the associated taxa clustered together in the bootstrap test (1,000 replicates) are shown next to the branches. Scale bar indicates nucleotide substitutions per site. CHA, China; HKN, Hong Kong; JPN, Japan; RUS, Russia; SKR or KOR, South Korea; VN, Vietnam.

The outbreak quickly spread nationwide across a large distance. This rapid spread occurred for several reasons: 1) the first infection was in a pig-farming complex, and pigs excrete the virus in large amounts; 2) detection of the first infection was delayed; 3) FMDV-contaminated feces from the index pig-farming complex was moved to other provinces to be recycled for use as fuel on November 17, before the first outbreak; 4) the virus has increased stability during the winter months, enabling it to be transmitted more easily; 5) culling of infected animals was not implemented quickly enough by affected farms; and 6) the distance between farms in the area was small.

The FMD virus is believed to have entered South Korea around November 9–16, 2010; the first clinical signs in pigs appeared on November 23, and serologic investigation found that the time point for FMD infection was November 14. The virus might have been brought into the country as a result of a farmer’s trip to Southeast Asia in early November.

FMDV isolates from Mongolia, Vietnam, and other countries in Asia largely group into 2 phylogenetic clusters on the basis of nucleotide similarities (*1*). To determine the relationship between the South Korea virus strain and those from other countries in Asia, we analyzed the viral protein 1 nucleotide sequence of an FMDV virus isolate from the first FMD case, in November 2010. The sequence showed >99% identity with the O serotype; this type also matched those found in Gyeonggi Province and another location in Gyeongbuk Province during December 2010. However, a group of FMD viruses identified in South Korea and People’s Republic of China (group 1) showed 6 amino acid residues of viral protein 1 different from those of other seasons or countries (Table 2). In addition, among other FMD outbreaks identified in neighboring countries, viruses that originated in China had the most similar composition in amino acid residues to those from South Korea (Table 2; Figure 2) (*1*,*9*).

**Table 2:** Comparison of VP1 amino acids of foot-and-mouth disease isolates from South Korea versus viruses originating in other countries in Southeast Asia, 2010

Group and strain	Country	Region and province	Date of collection	Similarities of VP1, %			Alignment of major differences in VP1 amino acids by position						Genbank accession no.
nt	aa	58	139	141	152	157	184
I													
O/Andong/SKR/2010	South Korea	Andong, Gyeongbuk	Nov 28	Ref	Ref		S	S	P	Q	R	T	JQ070321
O/PJ/SKR/2010	South Korea	Paju, Gyeonggi	Dec 15	99.22	100.0		–	–	–	–	–	–	This study
O/YC/SKR/2010	South Korea	Yeoncheon, Gyeonggi	Dec 15	99.22	100.0		–	–	–	–	–	–	This study
O/PC/SKR/2010	South Korea	Pyeongchang, Gangwon	Dec 21	99.06	99.53		–	–	–	–	–	–	This study
O/GH/SKR/2010	South Korea	Ganghwa, Incheon	Dec 24	99.22	100.0		–	–	–	–	–	–	This study
O/BY/CHA/2010	China	Shenzhen, Guangdong	Mar 4	98.75	100.0		–	–	–	–	–	–	JN998085
O/CHA/31/2010	China	NA	Feb 22	98.90	100.0		–	–	–	–	–	–	JF792356
II													
O/DY/CHA/2010	China	NA	NA	97.65	97.18		–	P	–	–	–	–	HQ652078
O/RUS/Jul 2010	Russia	Abagaytuy, Zabajkal’skijkray	Jul	99.06	99.06		–	P	–	–	–	–	JQ070329
III													
O/HKN/7/2010	China	Hong Kong	Feb 22	99.06	99.53		P	–	–	–	–	–	JQ070303
O/HKN/9/2010	China	Hong Kong	Feb 24	98.9	99.53		P	–	–	–	–	–	JQ070304
IV													
O/JPN/MZ1/2010	Japan	Miyazaki	May	98.90	99.06		–	–	–	P	–	A	AB618503
O/TZ/CHA/2010	China	NA	NA	98.44	99.06		–	–	–	P	–	A	HQ652081
V													
O/VN/YB08/2010	Vietnam	Yen Bai	Feb	98.28	99.06		P	–	T	–	–	–	HQ26078
O/GZ/CHA/2010	China	NA	Mar	98.59	98.12		P	–	T	–	–	–	JN998086
O/NC/CHA/2010	China	NA	NA	97.97	98.59		P	–	T	–	–	–	HQ652080
O/MY/CHA/2010	China	NA	NA	98.59	99.06		P	–	T	–	–	–	HQ652079
VI													
O/KOR/1/2010	South Korea	Ganghwa, Incheon	Apr 8	98.44	99.06		P	–	–	–	W	–	HM143846
O/KOR/10/2010	South Korea	Cheonyang, Chungnam	Apr 30	98.44	98.59		P	–	S	–	W	–	This study
O/KOR/11/2010	South Korea	Cheonyang, Chungnam	May 6	98.44	98.59		P	–	S	–	W	–	This study

*Groups are based on major differences in amino acids. VP1, viral protein 1; nt, nucleotides; aa, amino acids; SKR or KOR, South Korea; ref, referent; –, no difference; CHA, China; NA, not available; RUS, Russia; HKN, Hong Kong; JPN, Japan; VN, Vietnam.

## Conclusions

An outbreak of FMD in South Korea during November 2010–April 2011 was caused by serotype O FMDV and affected ≈3,700 farms; 153 farms were identified as index locations for new outbreaks. A total of 3.48 million susceptible animals were culled, including cattle and pigs. A vaccination program was effective in controlling the outbreak, and FMD incidence declined rapidly after its completion.

Technical Appendix Timeline of foot-and-mouth disease case detection and animals or farms requiring culling and cumulative numbers of culled animals or farms during foot-and-mouth disease outbreak, South Korea, 2012–2011.

## References

[1] Knowles NJ, He J, Shang Y, Wadsworth J, Valdazo-Gonzalez B, Onosato H, Southeast Asian foot-and-mouth disease viruses in Eastern Asia. Emerg Infect Dis. 2012;18:499–501 . 10.3201/eid1803.110908 22377196 PMC3309575

[2] Joo YS, An SH, Kim OK, Lubroth J, Sur JH. Foot-and-mouth disease eradication efforts in the Republic of Korea. Can J Vet Res. 2002;66:122–4 . 11989734 PMC226994

[3] Shin JH, Sohn HJ, Choi KS, Kwon BJ, Choi CU, Kim JH, Identification and isolation of foot-and-mouth disease virus from primary suspect cases in Korea in 2000. J Vet Med Sci. 2003;65:1–7. 10.1292/jvms.65.1 12576697

[4] Wee SH, Yoon H, More SJ, Nam HM, Moon OK, Jung JM, Epidemiological characteristics of the 2002 outbreak of foot-and-mouth disease in the Republic of Korea. Transbound Emerg Dis. 2008;55:360–8. 10.1111/j.1865-1682.2008.01045.x 18786074

[5] Park JH, Park JY, Kim YJ, Oem JK, Lee KN, Kye SJ, Vaccination as a control measure during the outbreak of foot-and-mouth disease in 2000 in Korea. Dev Biol (Basel). 2004;119:63–70 . 15742619

[6] Park JH, Lee KN, Ko YJ, Kim SM, Lee HS, Park JY, Diagnosis and control measures of the 2010 outbreak of foot-and-mouth disease A type in the Republic of Korea. Transbound Emerg Dis. 2012 May 27. Epub ahead of print. 10.1111/j.1865-1682.2012.01333.x 22630568

[7] Yoon H, Yoon SS, Wee SH, Kim YJ, Kim B. Clinical manifestations of foot-and-mouth disease during the 2010/2011 epidemic in the Republic of Korea. Transbound Emerg Dis. 2012;59:517–25. 10.1111/j.1865-1682.2011.01304.x 22273469

[8] Madhanmohan M, Nagendrakumar SB, Kumar R, Anilkumar J, Manikumar K, Yuvaraj S, Clinical protection, sub-clinical infection and persistence following vaccination with extinction payloads of O(1) Manisa foot-and-mouth disease monovalent vaccine and challenge in goats and comparison with sheep. Res Vet Sci. 2012;93:1050–9. 10.1016/j.rvsc.2011.10.006 22079173

[9] Muroga N, Hayama Y, Yamamoto T, Kurogi A, Tsuda T, Tsutsui T. The 2010 foot-and-mouth disease epidemic in Japan. J Vet Med Sci. 2012;74:399–404. 10.1292/jvms.11-0271 22075710

