# Supplementary material for: Control of Foot-and-Mouth Disease during 2010–2011 Epidemic, South Korea
Source: Emerg Infect Dis. 2013 Apr;19(4):655–9. doi: 10.3201/eid1904.121320 (PMC3647416; doi:10.3201/eid1904.121320)
Supplement: Technical Appendix — Timeline of foot-and-mouth disease case detection and animals or farms requiring culling and cumulative numbers of culled animals or farms during foot-and-mouth disease outbreak, South Korea, 2012–2011. [file 12-1320-Techapp-s1.pdf]

Article DOI: http://dx.doi.org/10.3201/eid1904.121320

Control of Foot-and-Mouth Disease during 2010–2011 Epidemic, South Korea

Technical Appendix

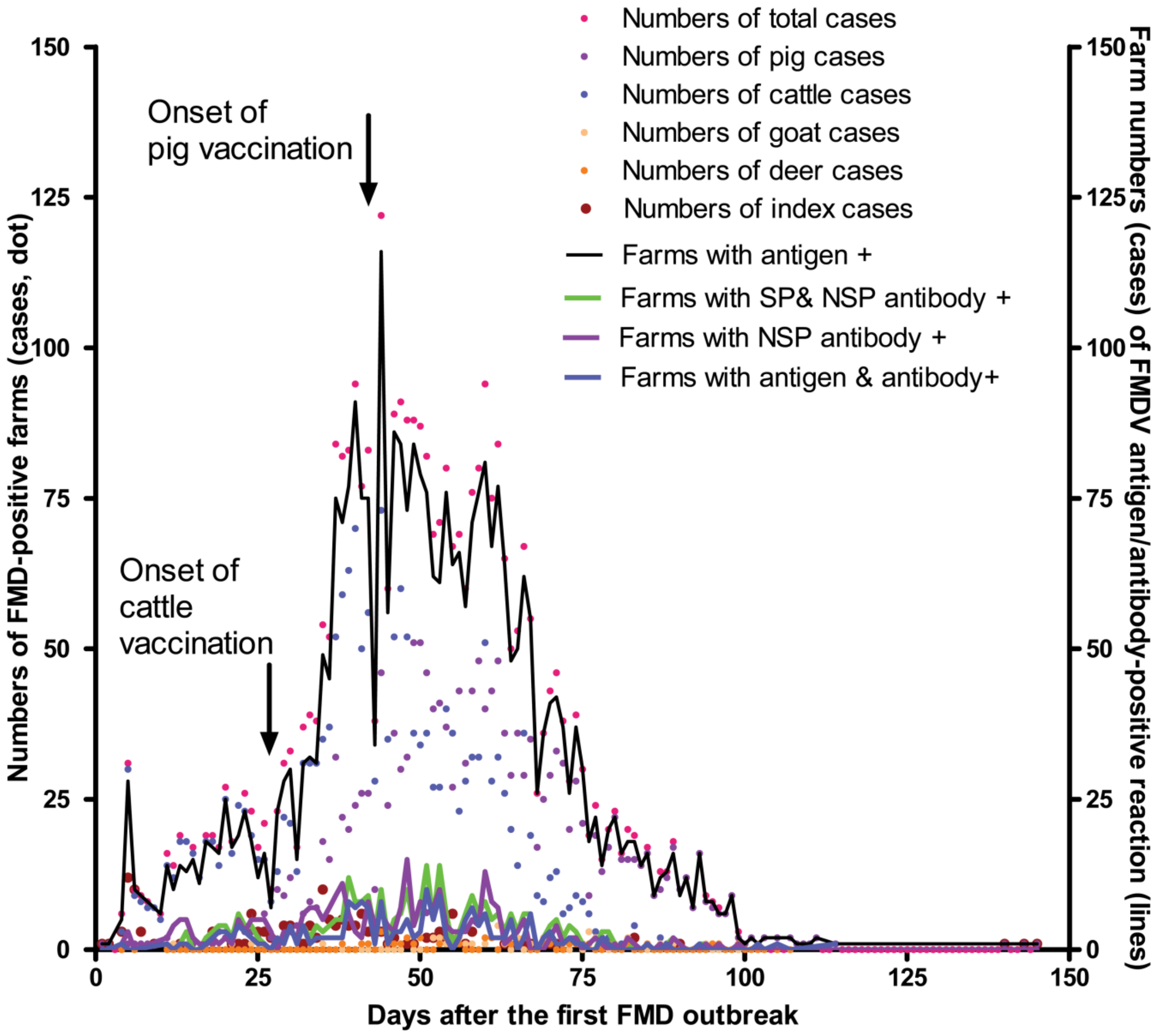

Technical Appendix Figure 1. Timeline of foot-and-mouth disease cases and case detection by foot-and-mouth disease virus antibody testing (structural and nonstructural protein analyses) for outbreak in South Korea, 2010–2011. Onset dates for vaccination of cattle and pigs are indicated.

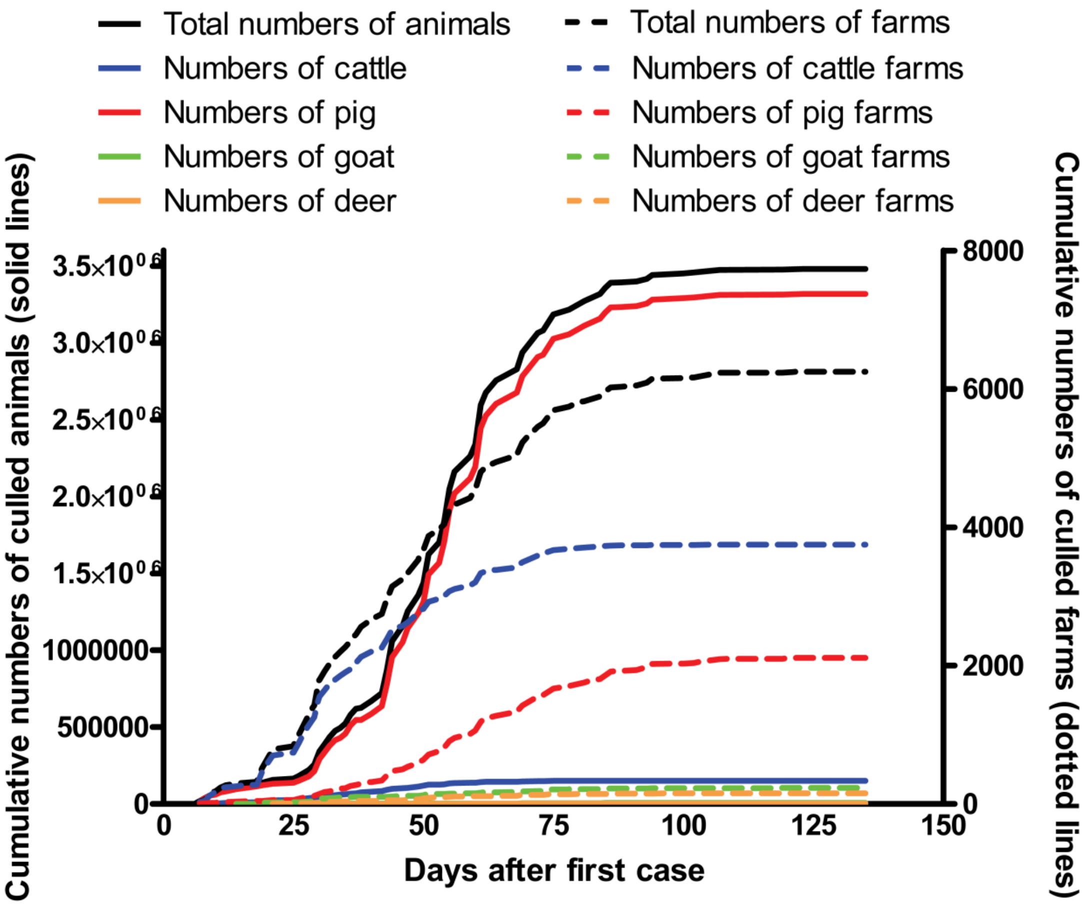

Technical Appendix Figure 2. Cumulative numbers of culled animals or farms during foot-and-mouth disease outbreak, South Korea, 2012–2011.

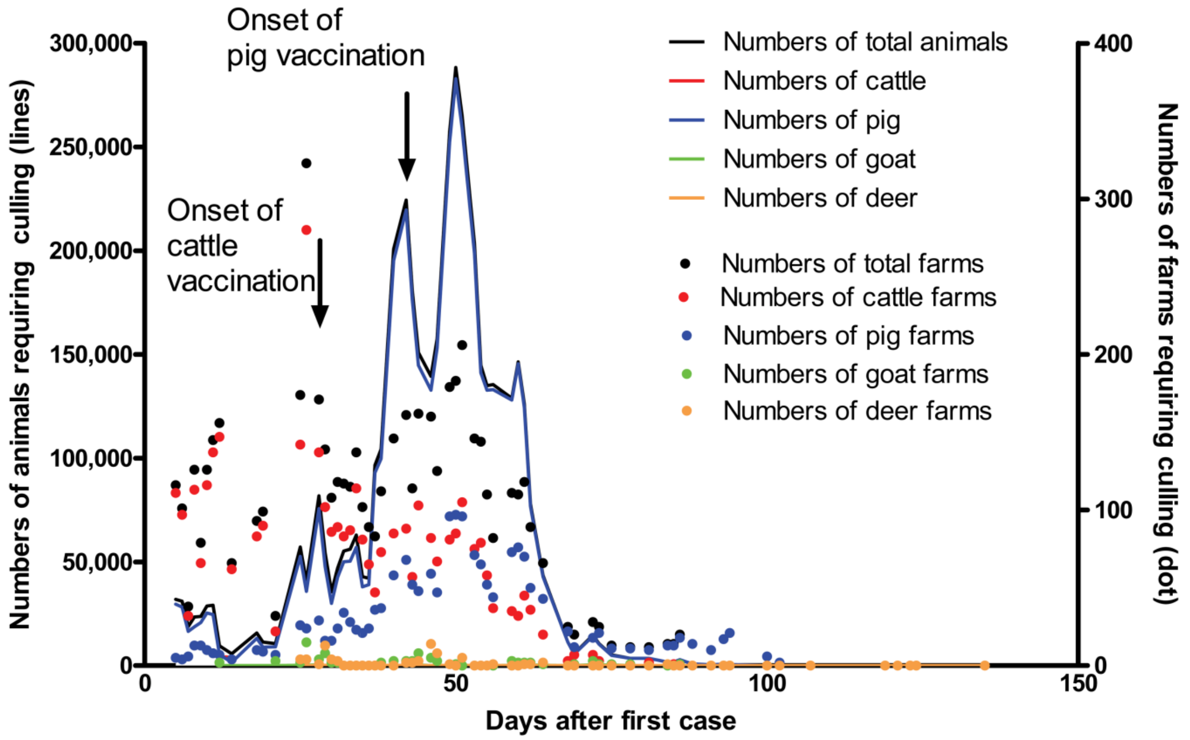

Technical Appendix Figure 3. Timeline of animals or farms requiring culling during foot-and-mouth disease outbreak, South Korea, 2010–2011. Onset dates for vaccination of cattle and pigs are indicated. Most culling occurred during January 2011; vaccination was completed by January 31, 2011.
